# Brd4 Inactivation Increases Adenoviral Delivery of BMP2 for Paracrine Stimulation of Osteogenic Differentiation as a Gene Therapeutic Concept to Enhance Bone Healing

**DOI:** 10.1002/jbm4.10520

**Published:** 2021-06-23

**Authors:** Christopher R Paradise, Rodolfo E De La Vega, M Lizeth Galvan, Margarita E Carrasco, Roman Thaler, Andre J van Wijnen, Amel Dudakovic

**Affiliations:** ^1^ Department of Orthopedic Surgery Mayo Clinic Rochester MN USA; ^2^ Center for Regenerative Medicine Mayo Clinic Rochester MN USA; ^3^ Musculosketal Gene Therapy Research Laboratory, Rehabilitation Medicine Research Center Mayo Clinic Rochester MN USA; ^4^ Department cBITE, MERLN Institute for Technology‐Inspired Regenerative Medicine Maastricht University Maastricht The Netherlands; ^5^ Department IBE, MERLN Institute for Technology‐Inspired Regenerative Medicine Maastricht University Maastricht The Netherlands; ^6^ Department of Biochemistry and Molecular Biology Mayo Clinic Rochester MN USA

**Keywords:** ADENOVIRUS, BMP2, BRD4, EPIGENETICS, OSTEOGENESIS, VIRAL THERAPY

## Abstract

Bromodomain (BRD) proteins are histone code interpreters that recognize acetylated lysines and link the dynamic state of chromatin with the transcriptional machinery. Here, we demonstrate that ablation of the Brd4 gene in primary mouse bone marrow–derived mesenchymal stem cells via a conditional Brd4^fl/fl^ allele suppresses osteogenic lineage commitment. Remarkably, loss of Brd4 function also enhances expression of genes in engineered adenoviral vectors, including Cre recombinase and green fluorescent protein (GFP). Similarly, vector‐based expression of BMP2 mRNA and protein levels are enhanced upon Brd4 depletion in cells transduced with an adenoviral vector that expresses BMP2 (Ad‐BMP2). Importantly, Brd4 depletion in MC3T3‐E1 and human adipose‐derived mesenchymal stem cells (AMSCs) transduced with Ad‐BMP2 enhances osteogenic differentiation of naïve MC3T3‐E1 cells via paracrine mechanisms based on transwell and conditioned medium studies. Our studies indicate that Brd4 depletion enhances adenoviral transgene expression in mammalian cells, which can be leveraged as a therapeutic strategy to improve viral vector‐based gene therapies. © 2021 The Authors. *JBMR Plus* published by Wiley Periodicals LLC on behalf of American Society for Bone and Mineral Research.

## Graphical abstract

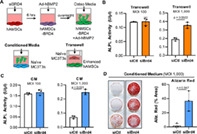

## Introduction

There is great need for improvement of outcomes in complicated orthopedic scenarios such as fracture nonunion and large bone defects.^(^ ^1^, ^2^ ^)^ Autogenous bone grafts, the clinical gold standard, are in limited supply and are associated with donor site morbidity.^(^ ^3^, ^4^ ^)^ Other available therapeutics may promote bone healing but also have several disadvantages including re‐fracture, inconsistent results, and failure to remodel.^(^ ^5^ ^)^ Among these, recombinant human bone morphogenetic protein 2 (rhBMP2) has been frequently studied because it is FDA‐approved for a limited number of clinical applications where formation of new bone is desired. Paradoxically, although BMP2 is highly osteo‐inductive, its clinical performance has disappointed.

As the only osteo‐inductive cytokine approved by the FDA for clinical use, rhBMP2 quickly became a popular treatment after its initial approval in 2002 for spinal fusion.^(^ ^6^, ^7^ ^)^ Since then, it has obtained regulatory approval for use in open tibial fractures and dental procedures, and it has become a widely used off‐label drug for recalcitrant bone lesions. However, clinical adoption has slowed due to efficacy, elevated costs, and reports of side effects.^(^ ^8^ ^)^ Because of these impediments, there is a compelling need for alternative strategies aimed at delivering lower doses of BMP2 for prolonged periods of time while increasing its efficacy.

BMP2 gene transfer using an adenoviral vector carrying the human BMP2 cDNA has been successfully used both in vivo and ex vivo for healing large bone defects in animal models.^(^ ^9^, ^10^, ^11^ ^)^ Yet, the use of high viral doses presents additional drawbacks including elevated costs and safety concerns. After the first reported lethal adverse event in a viral gene therapy clinical trial in 1999 and more recent lethal events,^(^ ^12^, ^13^ ^)^ safety has been a major consideration in the continued clinical development of viral gene transfer. Strategies with increased safety may emerge from reduced viral dosing while still achieving adequate transgene expression.

We have recently demonstrated that BMP2 dosing can be reduced in the presence of epigenetic enzyme inhibitors that prime chromatin for osteogenic stimuli.^(^ ^14^ ^)^ These studies evolved from earlier studies showing that posttranslational modification of histones H3 and H4 by acetylation or methylation supports skeletal development and osteogenic commitment and differentiation.^(^ ^15^, ^16^, ^17^, ^18^ ^)^ Extensive studies on enhancer of zeste homolog 2 (Ezh2), a methyltransferase that acts on histone 3 on lysine 27 (H3K27), revealed that it regulates skeletogenesis.^(^ ^19^, ^20^, ^21^, ^22^, ^23^, ^24^, ^25^ ^)^ Short‐term pharmacological inhibition of Ezh2 activity represents a strategy that can be applied to stimulate osteogenic differentiation in vitro and in vivo.^(^ ^14^, ^21^, ^26^, ^27^, ^28^, ^29^ ^)^ Mechanistically, inhibition of Ezh2 supports osteogenic commitment by reducing H3K27 tri‐methylation, an inhibitory epigenetic mark, which is dynamically replaced by the activating epigenetic mark H3K27ac.^(^ ^30^ ^)^

Because histone acetylation supports gene activation,^(^ ^31^, ^32^ ^)^ our current studies are focused on understanding the mechanism by which acetylation marks are recognized and interpreted in differentiating osteoblasts. Acetylated histones are bound by bromodomain (BRD) proteins that provide a direct link between the epigenetic landscape and transcriptional machinery.^(^ ^33^ ^)^ The bromodomain and extra‐terminal domain (BET) subfamily of BRD proteins is of particular interest as these epigenetic regulators can simultaneously engage with acetylated lysines via the BRD domains as well as transcriptional factors via the extra‐terminal domain.^(^ ^34^ ^)^ Recent studies by our group have shown that Brd4 and Brd2, two BET subfamily members, are required for osteogenic differentiation of MC3T3‐E1 pre‐osteoblasts and contribute to mechanical strain–mediated processes in osteoblastic SAOS‐2 sarcoma cells, respectively.^(^ ^30^, ^35^ ^)^ Recent studies support the idea that BET family members are key contributors of osteogenic differentiation.^(^ ^36^, ^37^ ^)^

In this study, we assess the contribution of Brd4 during osteogenic differentiation of primary bone marrow–derived mesenchymal stem/stromal cells (BMSCs). Our results show that Brd4 depletion via adenoviral Cre‐recombinase transfection of BMSCs that harbor the Brd4 conditional allele suppresses osteogenic differentiation. Furthermore, Brd4 depletion enhances adenoviral transgene expression, including human BMP2, in several cell culture models. Importantly, we demonstrate that this enhancement of BMP2 levels accelerates osteogenic differentiation of naïve MC3T3 pre‐osteoblast in the local environment via transwell and conditioned medium cultures.

## Materials and Methods

### Animal welfare

All studies utilizing mice were performed according to guidelines provided by the National Institutes of Health and the Institute of Laboratory Animal Resources, National Research Council. Our studies were approved by the Mayo Clinic Institutional Animal Care and Use Committee. Mice were housed in an accredited facility under a 12‐hour light/dark cycle and provided water and food (PicoLab Rodent Diet 20, LabDiet) *ad libitum*.

### Primary cell isolation and culture

Brd4^fl/fl^ mice utilized in our studies are a kind gift from Dr Anup Dey and Dr Keiko Ozato (National Institutes of Health, Bethesda, MD, USA).^(^ ^38^ ^)^ All mice are on the C57BL/6J genetic background and are genotyped as described by Lee and colleagues.^(^ ^38^ ^)^ BMSCs were isolated from 8‐ to 12‐week‐old Brd4^fl/fl^ and Brd4^wt/wt^ male and female mice as previously described.^(^ ^24^ ^)^ Briefly, bone marrow was flushed from femora and tibias and resulting cells were plated in 10 cm cell culture dishes in αMEM (Gibco/Thermo Fisher Scientific, Waltham, MA, USA) supplemented with 20% FBS (Atlanta Biologicals, Flower Branch, GA, USA), 1% antibiotic and antimycotic (Gibco/Thermo Fisher Scientific), and 1% non‐essential amino acids (Gibco/Thermo Fisher Scientific). After two media changes (6 days of culture), which removed all non‐adherent cell populations, BMSCs were detached by trypsin and replated in 6‐ and 12‐well plates at a density of 30,000 cells/cm^2^. One day later (day 0 of osteogenic differentiation), cells were transduced with Ad‐GFP‐Cre (University of Iowa Viral Vector Core Facility) and basal growth medium was supplemented with 50 μg/mL ascorbic acid, 10 mM beta glycerol phosphate, and 10^−8^ M dexamethasone to induce osteogenic differentiation. The initial osteogenic medium was replaced 4 days later and subsequent osteogenic medium changes occurred every 3 days. Western blotting analysis was done on protein lysates collected on day 4, RT‐qPCR analysis was performed on samples collected on days 4 and 25, MTS assay was performed on day 7, whereas Alizarin red staining occurred on day 25 of BMSC osteogenic differentiation.

### Ad‐GFP‐Cre transduction and +JQ1 treatment of MC3T3‐E1 cells

MC3T3‐E1 sc4 (referred to as MC3T3 or MC3T3‐E1) cells were maintained in αMEM with no ascorbic acid (Gibco/Thermo Fisher Scientific) supplemented with 10% fetal bovine serum (FBS) (Atlanta Biologicals), 100 U/mL penicillin, and 100 μg/mL streptomycin (Gibco/Thermo Fisher Scientific). Cells were seeded in 12‐well plates at a density of 10,000 cells/cm^2^ and allowed to adhere overnight. The next day, cultures were transduced with Ad‐GFP‐Cre in the presence of vehicle (DMSO) and several +JQ1 (Cayman Chemical, Ann Arbor, MI, USA) concentrations. Four days later, GFP fluorescent images were captured on an Axio Vert.A1 microscope using an AxioCam ICc 5 digital camera (Zeiss, Thornwood, NY, USA) and cells were then lysed and protein levels assessed by Western blotting analysis.

### Transduction of MC3T3‐E1 cells with Ad‐BMP2

MC3T3‐E1 cells were plated in 24‐well plates and 24‐well 0.4 μm polyester membrane cell culture inserts (Corning Inc., Corning, NY, USA) in maintenance medium (10,000 cells/cm^2^) and allowed to adhere. The next day, a cohort of cells grown in 24‐well plates was transfected with 20 nM smart‐pool control and mouse Brd4 siRNAs (Dharmacon/GE Healthcare, Lafayette, CO, USA) by Lipofectamine RNAiMAX (Thermo Fisher Scientific). Six hours later, the same batch of cells was transduced with BMP2 adenovirus (Ad‐BMP2) in serum‐free MEM alpha medium followed by serum reintroduction 2 hours later. A first‐generation adenovirus carrying the human BMP2 cDNA under the transcriptional control of the cytomegalovirus promoter was used for transduction, as previously reported.^(^ ^39^ ^)^ The next day (day 0 of osteogenic differentiation), growth medium was replaced with osteogenic medium (growth medium supplemented with 50 μg/mL ascorbic acid 10 mM beta glycerol phosphate) in transfected/transduced cultures.

#### Co‐culture experiment

Naïve cells grown in cell culture inserts (top cell layer) were transferred to transfected/transduced cell cultures (bottom cell layer) on day 0 of osteogenic differentiation. Differentiation media was replaced on days 2 and 5. Alkaline phosphatase activity assay and RT‐qPCR analysis were performed with naïve cells grown in transwells on day 8 of osteogenic differentiation.

#### Conditioned media experiment

On day 0, osteogenic differentiation was induced in a naïve cohort of MC3T3 cells grown in 12‐well plates. Two days later, conditioned media from transfected/transduced cells at a 1:1 ratio (50% conditioned media and 50% fresh osteogenic media) was added to naïve cells. The conditioned media regimen was repeated on day 5 of osteogenic differentiation. Alkaline phosphatase activity assay on conditioned naïve cells was performed on day 8 of osteogenic differentiation.

### siGLO transfection

siGLO green transfection indicator (0.02 μM) (Horizon, D‐001630‐01‐05) was transfected into MC3T3‐E1 (passage 7) or hAMSC (passage 5) as described above using Lipofectamine RNAiMax Transfection Reagent (Thermo Fisher Scientific, 13778150). After 24 hours, cells were washed with 1x PBS and stained with Hoechst 3342 nuclear dye (Sigma, St. Louis, MO, USA; 94403) (0.5 μL/mL in PBS). Cells were imaged using the Axio Vert.A1 microscope using an AxioCam ICc 5 digital camera (Zeiss) and transfected cells (siGLO positive) and total number of cells (Hoechst positive) quantified by ImageJ software.^(^ ^40^ ^)^

### BMP2 quantification

Conditioned media from transfected and/or transduced cells was collected on days 2, 5, and 8, and 11 of osteogenic differentiation and stored at −80°C until all samples were available for analysis. Human BMP2 quantification in the medium was measured using a commercially available DuoSet ELISA kit (R&D Systems, Minneapolis, MN, USA). The assay was conducted following the manufacturer's protocol for each biological sample.

### Transduction of human stem cells with Ad‐BMP2

Clinically relevant human adipose‐derived mesenchymal stem cells (AMSCs), which have been extensively characterized,^(^ ^21^, ^41^, ^42^, ^43^, ^44^, ^45^ ^)^ were maintained in advanced minimum essential medium (Gibco/Thermo Fisher Scientific) supplemented with 5% human platelet lysate (Mill Creek Life Sciences, Rochester, MN, USA), 100 U/mL penicillin and 100 μg/mL streptomycin (Gibco/Thermo Fisher Scientific), 2 mM L‐glutamine (Gibco/Thermo Fisher Scientific), and 2 U/mL heparin (Baxter, Deerfield, IL, USA). At the time of the experiment, AMSCs were plated in 24‐well plates in maintenance medium (10,000 cells/cm^2^) and allowed to adhere. The next day, AMSCs were transfected with control and human BRD4 siRNA smart pools (Dharmacon/GE Healthcare), transduced with Ad‐BMP2, which was followed by platelet lysate reintroduction (same process as for MC3T3‐E1 cells). The next day (day 0 of osteogenic differentiation), AMSC growth medium was replaced with MC3T3 osteogenic medium. A switch to MC3T3 medium supplemented with 50 μg/mL ascorbic acid and 10 mM beta glycerol phosphate was selected to support osteogenic differentiation of MC3T3 that will be co‐cultured or exposed to AMSC conditioned medium. As with transfected/transduced MC3T3 cells, naïve MC3T3 were differentiated in co‐cultures (cell culture inserts) or in the conditioned medium of transfected/transduced AMSCs. Alkaline phosphatase activity (MC3T3 differentiated in co‐culture inserts and in conditioned medium) was performed on day 8, whereas Alizarin red staining (MC3T3 differentiated in conditioned medium) was done on day 23 of osteogenic differentiation.

### Gene expression analysis

Cells were lysed in TRI‐Reagent (Zymo Research, Irvine, CA, USA) and RNA was then isolated using the Direct‐zol RNA isolation kit (Zymo Research). After quantification and quality assessment using a NanoDrop 2000 spectrophotometer (Thermo Fisher Scientific), RNA samples were reverse transcribed into cDNA using the M‐MLV Reverse Transcriptase reagents and protocol (Promega, Madison, WI, USA). Gene expression was then quantified by real‐time quantitative PCR with QuantiTect SYBR Green PCR Kit (Qiagen, Valencia, CA, USA) and the CFX384 Real‐Time System (Bio‐Rad Laboratories, Hercules, CA, USA). Transcript levels were calculated using the 2^ΔΔCt^ method and normalized to the housekeeping gene Gapdh, which was set at 100. Primer pairs utilized in this study are provided (Supplemental Table S1).

### Western blotting

Whole‐cell proteins were extracted using radioimmunoprecipitation buffer (150 mm NaCl, 50 mm Tris, pH 7.4, 1% sodium deoxycholate, 0.1% sodium dodecyl sulfate, 1% Triton X‐100) complemented with protease inhibitor cocktail (Sigma) and phenylmethylsulfonyl fluoride (Sigma). Lysates were cleared by centrifugation, protein concentrations determined by the DC Protein Assay (Bio‐Rad), and equal amounts of protein for each sample were resolved using SDS‐PAGE gels and transferred to polyvinylidene difluoride membranes. After blocking in 5% nonfat dry milk (1 hour at room temperature), primary antibodies (overnight at 4°C) and secondary antibodies (1 hour at room temperature) were added sequentially. Protein signal was visualized by SuperSignal West Femto Reagent (Thermo Fisher Scientific) and the ChemiDoc Imaging System (Bio‐Rad). Primary antibody information: Brd4 (Bethyl Laboratories, Montgomery, TX, USA; cat. #A301‐985A; 1:2000), Gapdh (Cell Signaling Technology, Danvers, MA, USA; cat. #51745; 1:5000), GFP (Cell Signaling; cat. #2555S, 1:1000), and Cre (Cell Signaling; cat. #12830S; 1:1000).

### MTS activity assay

Metabolic activity of cell cultures was assessed by [3‐(4,5‐dimethylthiazol‐2‐yl)‐5‐(3‐carboxymethoxyphenyl)‐2‐(4‐sulfophenyl)‐2H‐tetrazolium (MTS) activity assay (Promega) as suggested by the manufacturer. Enzymatic activity was determined at 490 nm using a SpectraMAX Plus spectrophotometer (Molecular Devices, San Jose, CA, USA).

### Alkaline phosphatase activity assay

Differentiation medium was removed, cell cultures washed with PBS, followed by addition of Tris‐EDTA buffer (0.1x). The plates were then stored at −80°C for at least 2 hours followed by thawing at room temperature. Para‐nitrophenylphosphate solution (2.5 mg 4‐nitrophenylphosphate disodium salt hexahydrate [Sigma] per 1 mL of buffer [0.1 M diethanolamine, 150 mM NaCl, 2 mM MgCl_2_]) was then added to each well. After a 30‐minute incubation at room temperature, absorbance was measured at 405 nm using the SpectraMAX Plus spectrophotometer (Molecular Devices). Raw values were then fit to a standard curve that was generated using reconstituted alkaline phosphatase enzyme (Roche, Indianapolis, IN, USA) to determine relative enzymatic activity within cultures.

### Alizarin red staining

At the end of osteogenic differentiation, media was aspirated, cultures washed with PBS, and cells fixed in 10% neutral buffered formalin (NBF). NBF was removed 1 hour later, cells washed with PBS, and the cultures stained for 10 minutes with 2% Alizarin red (Thermo Fisher Scientific). After removal of Alizarin red, cultures were washed with water five times. Plates were scanned and staining intensity was quantified by ImageJ software.^(^ ^40^ ^)^

### Statistics

GraphPad Prism version 8.2.1 for Windows (GraphPad, La Jolla, CA, USA) was used for graphing and statistical analysis. Continuous variables were analyzed by one‐way ANOVA and unpaired Student *t* test. When applicable, statistical significance is noted in the figures.

## Results

### Brd4 depletion inhibits osteogenic differentiation of mesenchymal stem cells

**Fig. 1:**
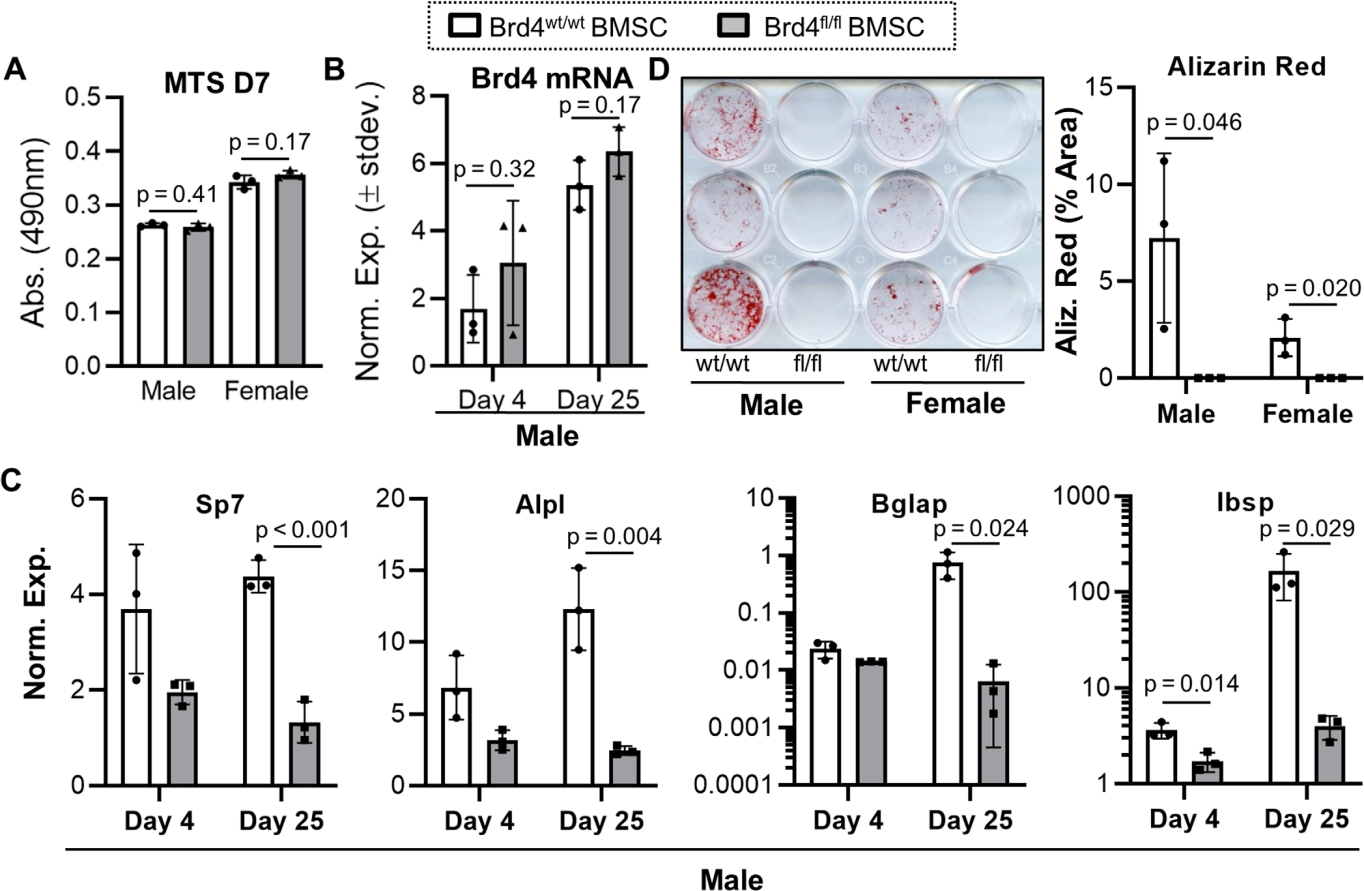
Brd4 depletion inhibits osteogenic differentiation of mouse BMSCs. BMSCs derived from male and female Brd4^wt/wt^ and Brd4^fl/fl^ mice were transduced with Ad‐GFP‐Cre and differentiated into osteogenic lineage. Days represent time after transduction and osteogenic induction. MTS assay conducted on day 7 for male‐ and female‐derived cells (*n* = 3, mean ± standard deviation) (*A*). RT‐qPCR analysis for Brd4 (*B*) and osteogenic genes (*C*) on days 4 and 25 in male‐derived cells (*n* = 3, mean ± standard deviation). Alizarin red staining and quantification performed on day 25 of male‐ and female‐derived cell cultures (*n* = 3, mean ± standard deviation). Statistical significance is indicated in the figure panels.

To determine the contribution of Brd4 to osteogenic commitment of primary mesenchymal progenitor cells, we transduced BMSCs from Brd4^wt/wt^ and Brd4^fl/fl^ mice with a GFP and Cre recombinase adenovirus (Ad‐GFP‐Cre) and differentiated cells into the osteogenic lineage by addition of ascorbic acid, β‐glycerol phosphate, and dexamethasone (Fig. 1). In Brd4^fl/fl^ cells, loxP sites flank the ATG protein translational start site within exon 3 of the Brd4 gene.^(^ ^46^ ^)^ Cre recombinase activity is designed to render the gene dysfunctional by inhibiting protein translation of Brd4, while preserving mRNA transcription.

**Fig. 2:**
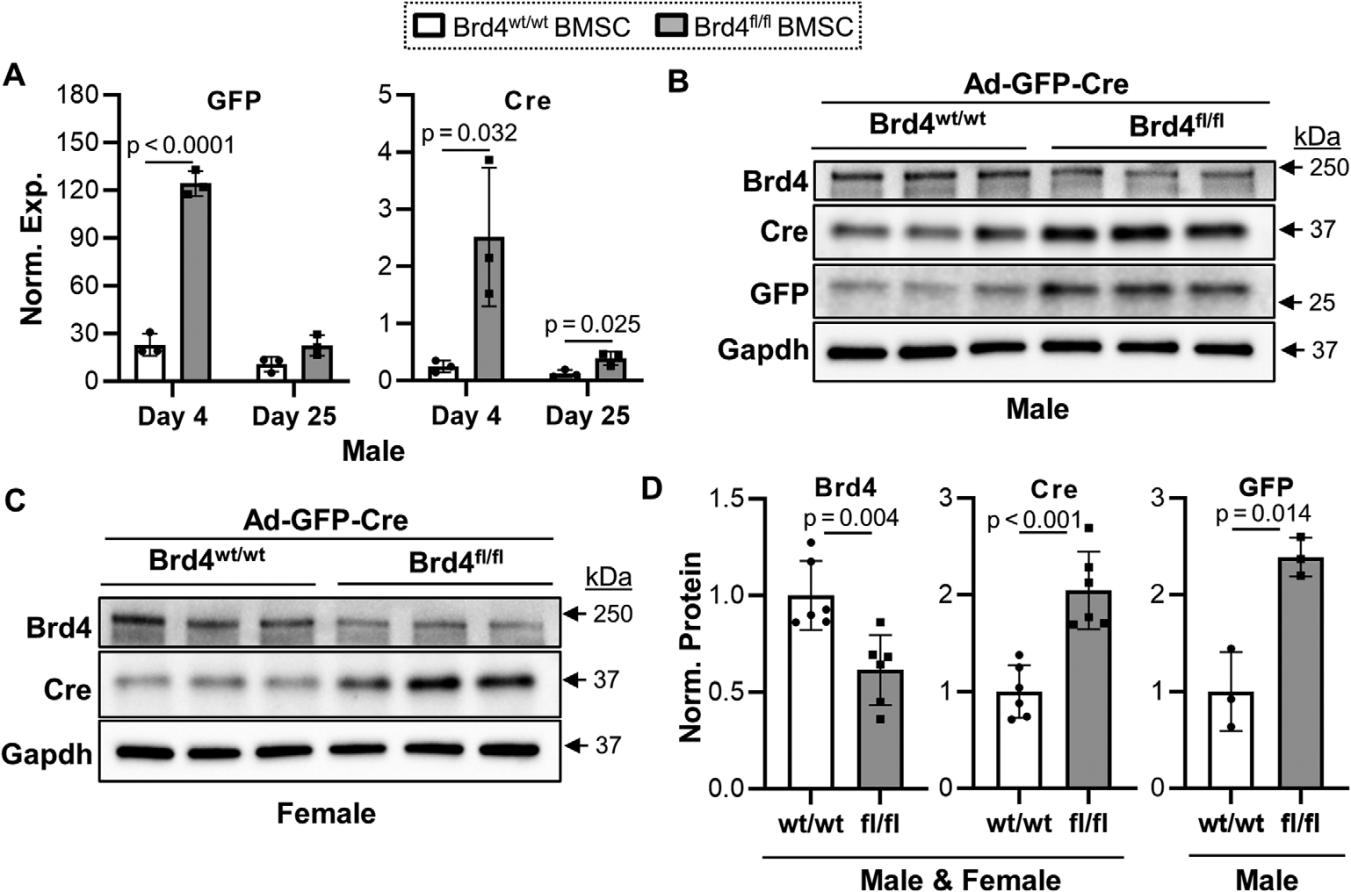
Brd4 depletion enhances expression of adenoviral transgenes in mouse BMSCs. BMSCs derived from male and female Brd4^wt/wt^ and Brd4^fl/fl^ mice were transduced with Ad‐GFP‐Cre and differentiated into osteogenic lineage. Days represent time after transduction and osteogenic induction. RT‐qPCR analysis for GFP and Cre, which are adenoviral transgenes, on days 4 and 25 in male‐derived cells (*n* = 3, mean ± standard deviation) (*A*). Western blot analysis for Brd4, GFP, Cre, and Gapdh performed on protein lysates collected on day 4 from male‐ (*B*) and female‐derived (*C*) cells. Combined quantification of Western blots performed in male‐ and female‐derived cells (*n* = 6 for Brd4 and Cre, *n* = 3 for GFP [male only], mean ± standard deviation). Quantification of statistical significance is indicated in the figure panels.

Cell viability, as measured by MTS activity assay, is similar between Brd4^wt/wt^ and Brd4^fl/fl^ groups 7 days after Ad‐GFP‐Cre transduction and osteogenic induction in both male‐ and female‐derived BMSCs (Fig. 1*A*). A direct comparison of metabolic activity between sexes is not feasible as MTS activity assays were done independently for male‐ and female‐derived cells. In addition to similar metabolic activity, Brd4 mRNA levels are comparable between Brd4^wt/wt^ and Brd4^fl/fl^ BMSCs on days 4 and 25 of osteogenic differentiation (Fig. 1*B*). Transcription of Brd4 mRNA appears to trend higher in the Brd4^fl/fl^ group at both days and may suggest a compensatory response to a reduction in Brd4 protein levels (Fig. 2). Importantly, expression levels of key osteoblast differentiation markers, including osterix/Sp7, alkaline phosphatase/Alpl, osteocalcin/Bglap, and bone sialoprotein/Ibsp, are suppressed in BMSCs derived from Brd4^fl/fl^ male mice (Fig. 1*C*). Similar Brd4 and osteogenic gene expression patterns are also observed in female BMSC cohorts (data not shown). Corroborating the RT‐qPCR analysis, Alizarin red staining of BMSC cultures on day 25 of osteogenic differentiation demonstrates a significant reduction in mineral deposition upon Ad‐GFP‐Cre infection in Brd4^fl/fl^ cohorts (Fig. 1*D*). Similar mineralization results are obtained for both sexes. Taken together, these data show that adenoviral Cre transduction inhibits osteogenic differentiation of Brd4^fl/fl^ mouse BMSCs. Our current findings complement our previous studies showing that Brd4 inhibition and depletion also inhibit maturation of pre‐committed MC3T3‐E1 pre‐osteoblasts.^(^ ^30^ ^)^

### Brd4 loss enhances adenoviral transgene mRNA and protein levels in BMSCs

To assess adenoviral transduction of Brd4^wt/wt^ and Brd4^fl/fl^ BMSCs, we monitored expression of GFP and Cre recombinase by RT‐qPCR and Western blot analyses (Fig. 2). Although the same viral load was used for both Brd4 genotypes, GFP and Cre expression is significantly elevated in BMSCs derived from Brd4^fl/fl^ mice on day 4 (Fig. 2*A*). We note that expression differences for GFP and Cre between Brd4^wt/wt^ and Brd4^fl/fl^ BMSCs are curbed on day 25, indicating a transient induction of viral transgenes. While Ad‐GFP‐Cre adenoviral transduction did not decrease Brd4 mRNA levels (Fig. 1*B*), a reduction in protein levels is observed due to start codon inactivation in BMSCs from Brd4^fl/fl^ mice when compared with Brd4^wt/wt^ BMSCs that are refractory to Cre recombination (Fig. 2*B, C*). Analogous results were obtained with BMSCs from either female or male mice because inactivation of the Brd4 locus gene depends solely on Cre recombination and is not expected to be influenced by sex. Importantly, protein levels of adenoviral transgenes, GFP and Cre, are significantly enhanced in BMSCs that exhibit a reduction in Brd4 protein levels (ie, Brd4^fl/fl^ transduced with Ad‐GFP‐Cre). Together, our results demonstrate successful reduction in Brd4 protein in Brd4^fl/fl^ BMSCs upon Ad‐GFP‐Cre transduction but also suggest that depletion of endogenous Brd4 may enhance transcription of adenoviral transgenes in mammalian cells.

### The BET inhibitor +JQ1 enhances expression of adenoviral transgene in MC3T3 pre‐osteoblasts

**Fig. 3:**
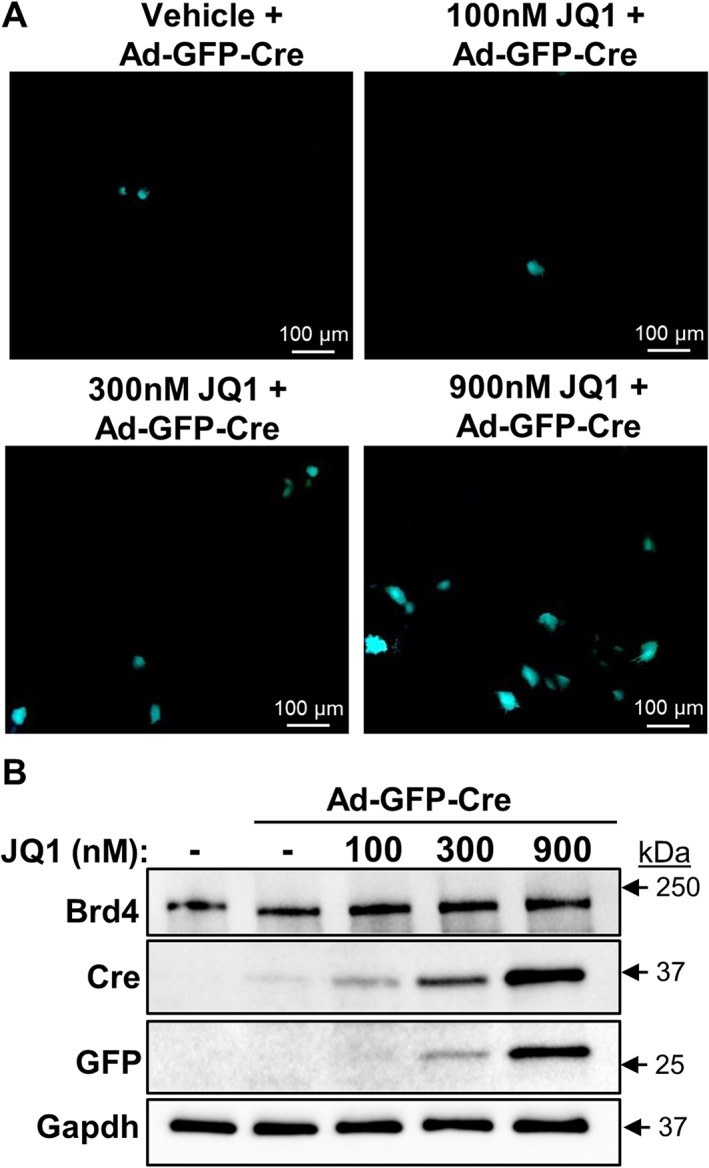
BET protein inhibition by +JQ1 enhances expression of adenoviral transgenes in MC3T3‐E1 cells. MC3T3‐E1 cells were transduced with Ad‐GFP‐Cre and treated with vehicle and several +JQ1 concentrations. GFP visualization by microscopy on day 4 (*A*). Western blot analysis for Brd4, GFP, Cre, and Gapdh performed on protein lysates collected on day 4 (*B*).

To further assess the role of Brd4 on adenoviral transgene expression, MC3T3‐E1 pre‐osteoblasts were transduced with Ad‐GFP‐Cre in the presence or absence of +JQ1, a small molecule inhibitor that binds competitively to bromo‐domains and prevents BET proteins from binding acetylated lysine residues.^(^ ^47^ ^)^ +JQ1 inhibits MC3T3‐E1 osteogenic differentiation at non‐toxic concentrations (100 nM).^(^ ^30^ ^)^ Similar to protein depletion, inhibition of Brd4 acetyl reader activity enhances adenoviral transgene expression in mammalian cells (Fig. 3). Fluorescence microscopy revealed robust GFP signals 4 days after transduction and concomitant vehicle or +JQ1 treatment (300 and 900 nM) (Fig. 3*A*). Similarly, Western blotting analysis reveals strong upregulation of adenoviral transgene proteins GFP and Cre with increasing concentration of +JQ1 (Fig. 3*B*). Collectively, our data reveal that inhibition of BET proteins by +JQ1 enhances the levels of adenoviral transgene proteins in transduced mammalian cells.

### Brd4 depletion enhances expression of adenoviral transgenic BMP2

**Fig. 4:**
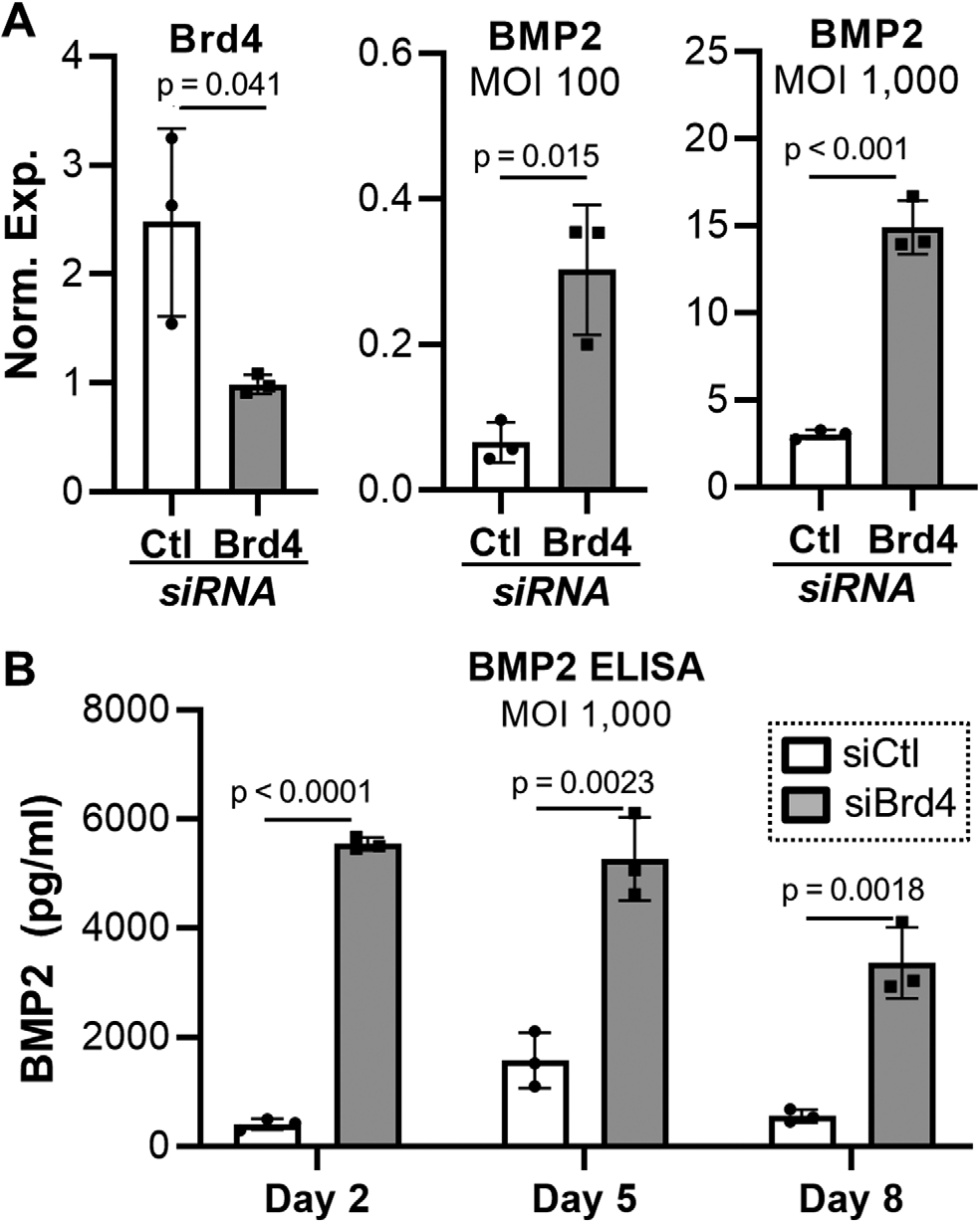
Brd4 depletion enhances expression of adenoviral BMP2 in MC3T3‐E1 cells. MC3T3‐E1 cells were transfected with control or Brd4 siRNA, transduced with Ad‐BMP2, and differentiated into osteogenic lineage. Days represent time after osteogenic induction. Expression of Brd4 (multiplicity of infection [MOI] = 1000) and BMP2 (MOI = 100 and 1000) on day 2 (*A*). Detection of BMP2 in medium by ELISA assay on days 2, 5, and 8 (MOI = 1000, *n* = 3, mean ± standard deviation) (*B*). Statistical significance is indicated in the figure panels.

**Fig. 5:**
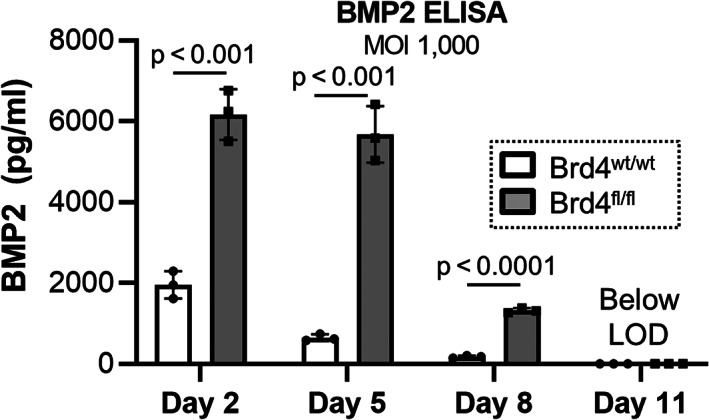
Brd4 depletion enhances expression of adenoviral BMP2 in mouse BMSCs. BMSCs were collected from 10‐week‐old Brd4^wt/wt^ and Brd4^fl/fl^ female mice. Cells were transduced with Ad‐Cre (multiplicity of infection [MOI] = 1000) and Ad‐BMP2 (MOI = 1000). Osteogenic differentiation was initiated 2 days after transduction (day 2). Media was collected at indicated time points and utilized for ELISA to quantify the level of human BMP2 (*n* = 3, mean ± standard deviation). LOD = limit of detection. Statistical significance is indicated in the figure.

Based on the enhanced expression of GFP and Cre as adenoviral transgene proteins observed upon Brd4 loss or +JQ1 treatment, we assessed whether siRNA depletion of Brd4 is a viable molecular strategy for enhancing human BMP2 expression delivered using an adenoviral vector (Fig. 4). To establish more precise cause‐and‐effect relationships, we opted for siRNA‐mediated Brd4 depletion rather than Brd4 blockade using BET inhibitors such as +JQ1, as these inhibitors also target other BET family members (eg, Brd2 and Brd3).^(^ ^48^ ^)^ Similar to induction of adenoviral GFP and Cre (Fig. 2), Brd4 depletion significantly enhances transcription of adenoviral BMP2 at 100 and 1000 multiplicity of infection (MOI) 2 days after siRNA transfection and adenoviral BMP2 transduction (Fig. 4*A*). Although BMP2 transcript expression levels are directly comparable between the two MOIs, the results are graphed using two separate bar charts for optimal data representation and visualization. To monitor protein levels, a human BMP2 ELISA assay was employed (Fig. 4*B*). Similar to GFP and Cre protein levels in BMSCs and MC3T3s (Figs. 2 and 3), the human BMP2 protein levels after adenoviral transduction are clearly elevated in the growth medium of MC3T3‐E1 cells in which Brd4 was depleted (Fig. 4*B*). Importantly, elevated BMP2 levels can be detected 8 days after siRNA‐mediated depletion of Brd4 and Ad‐BMP2 transduction. It is noteworthy that the enhanced BMP2 levels are transient as protein levels of this cytokine begin to drop off 8 days after transduction in Brd4 depleted cells. To further evaluate the timing and duration of the enhanced adenoviral transgene expression, we employed the primary Brd4^fl/fl^ BMSC culture model (Fig. 5). In these experiments, Brd4^fl/fl^ and Brd4^wt/wt^ BMSCs were dually transduced with Ad‐Cre and Ad‐BMP2. As with MC3T3 cells, Brd4 depletion (ie, Ad‐Cre transduction in Brd4^fl/fl^) significantly enhances BMP2 protein production 2, 5, and 8 days after transduction. Interestingly and similar to MC3T3 cells, BMP2 levels begin to decrease on day 8 and return to baseline (below limit of detection) by day 11 post‐transduction. Together, these data demonstrate that adenoviral expression and production of BMP2 can be enhanced by Brd4 depletion early in the culture period in both MC3T3 and BMSC cell culture models.

### Elevated BMP2 levels promote osteogenic differentiation by paracrine mechanisms

**Fig. 6:**
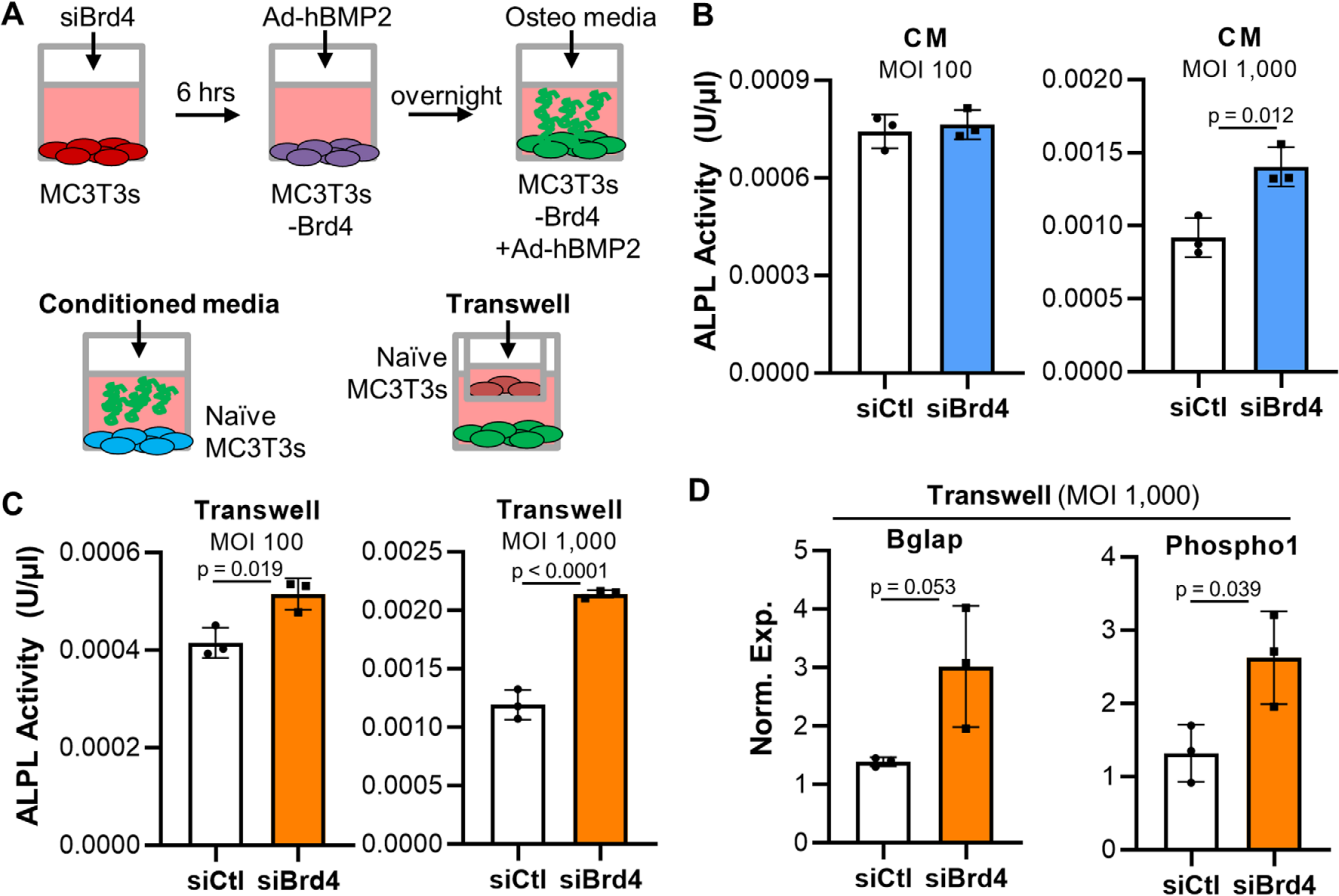
Brd4 depleted and Ad‐BMP2 transduced MC3T3‐E1 cells stimulate osteogenic differentiation within the local environment. MC3T3 cells were transfected with control or Brd4 siRNA, transduced with Ad‐BMP2, and exposed to osteogenic stimulus. Naïve MC3T3 cells were then differentiated in co‐cultures (transwell system) or conditioned medium (CM) derived from transfected/transduced MSC3T3 cells. The experimental design is shown (*A*). Alkaline phosphatase activity assay of naïve MC3T3 cells differentiated in the CM of transduced/transfected MSC3T3 cells (*B*) as well as together with transduced/transfected MC3T3 via transwells (*C*) on day 8 of osteogenic differentiation (*n* = 3, mean ± standard deviation). RT‐qPCR analysis for Bglap and Phospho1, which are osteogenic differentiation markers, in naïve MC3T3 differentiated together in transwell culture with transduced/transfected MC3T3 (*n* = 3, mean ± standard deviation) (*D*). MOI = multiplicity of infection, CM = conditioned medium. Statistical significance is indicated in the figure panels.

Increased production of adenoviral BMP2 by MC3T3 cells upon Brd4 depletion is predicted to induce osteogenic differentiation of BMP2‐responsive cells via paracrine mechanisms. Therefore, we assessed whether elevated BMP2 levels in conditioned media can stimulate osteogenic differentiation of naïve MC3T3 cells that were neither transfected with siRNAs nor transduced with Ad‐BMP2. We also note that MC3T3 cells do not express endogenous BMP2 mRNA based on RNA‐seq analysis of MC3T3 cells from multiple sources, albeit these cells express low mRNA levels for other BMP proteins (unpublished observations). To assess this, naïve MC3T3 cells were exposed to conditioned medium collected from MC3T3 cells transfected with Brd4 siRNA and transduced with Ad‐BMP2 using both transwell and conditioned medium assays (Fig. 6*A*). Our control data establish robust transfection efficiency and Brd4 knock‐down in transfected and transduced MC3T3 cells (Supplemental Fig. S1). Briefly, we observe ~60% reduction in Brd4 mRNA and ~ 75% reduction in Brd4 protein levels (Supplemental Fig. S1*A–C*). In support, cell counting for siGLO green transfection indicator and Hoechst reveals that most MC3T3 cells are successfully transfected in these experiments (Supplemental Fig. 1*D, E*). Alkaline phosphatase activity, a well‐established osteogenic marker, reveals that osteogenic differentiation of naïve MC3T3 cells is enhanced upon exposure to the medium of MC3T3 transduced with Ad‐BMP2 and reduced Brd4 levels in conditioned medium culture system at 1000 MOI (Fig. 6*B*) as well as transwell culture system at 100 and 1000 MOI (Fig. 6*C*). In support of the alkaline phosphatase activity assays, expression of key osteogenic markers, Bglap and phosphoethanolamine/phospho1, is also enhanced in differentiating naïve MC3T3 cells when cultured together with MC3T3 transfected with Brd4 siRNA and transduced with Ad‐BMP2 (Fig. 6*D*). These data demonstrate that enhanced adenoviral BMP2 expression by MC3T3 can mediate local paracrine stimulation of osteogenic differentiation of naïve MC3T3s in the cell culture environment.

**Fig. 7:**
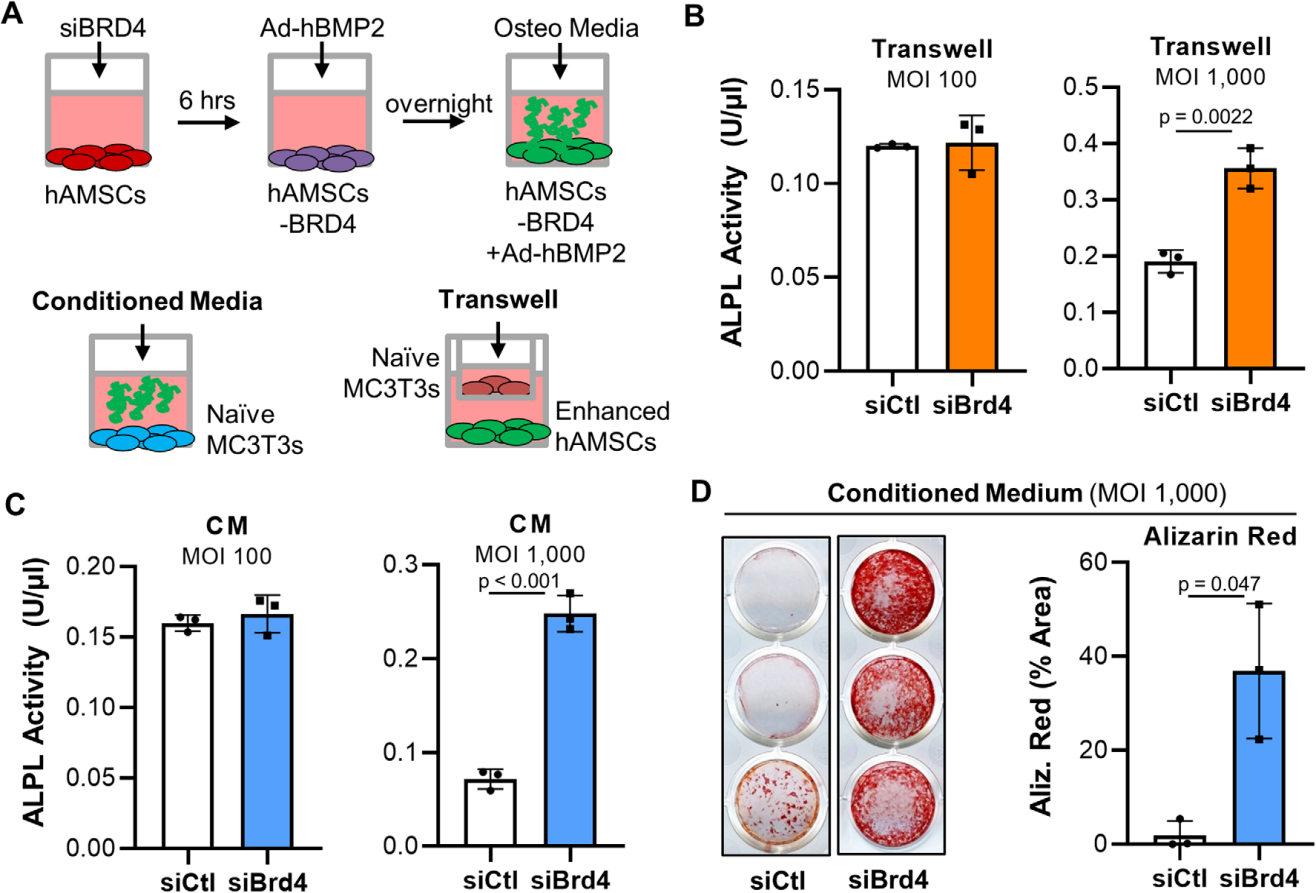
Brd4‐depleted and Ad‐BMP2‐transduced AMSCs stimulate osteogenic differentiation within the local environment. AMSCs were transfected with control or BRD4 siRNA, transduced with Ad‐BMP2, and exposed to osteogenic stimulus. Naïve MC3T3 cells were then differentiated in co‐cultures (transwell system) or conditioned medium (CM) derived from transfected/transduced AMSCs. The experimental design is shown (*A*). Alkaline phosphatase activity assay of naïve MC3T3 cells differentiated together with transduced/transfected AMSCs via transwells (*B*) and in the CM of transduced/transfected AMSCs (*C*) on day 8 of osteogenic differentiation (*n* = 3, mean ± standard deviation). Alizarin red staining and quantification of naïve MC3T3 differentiated in the CM of transduced/transfected AMSCs (*n* = 3, mean ± standard deviation) (*D*). MOI = multiplicity of infection, CM = conditioned medium. Statistical significance is indicated in the figure panels.

To enhance the clinical relevance of our findings, we also examined whether BRD4 inhibition enhances adenovirus BMP2 production in primary human AMSCs that are currently considered for different regenerative therapies.^(^ ^41^, ^43^, ^45^, ^49^ ^)^ Parallel to studies above, these investigations evaluated whether conditioned media containing adenoviral BMP2 that was enhanced by BRD4 depletion could enhance differentiation of pre‐committed naïve MC3T3 osteoblasts (Fig. 7*A*). As with MC3T3 cells, our control data establish robust transfection efficiency and significant BRD4 knock‐down in transfected and transduced AMSCs (Supplemental Fig. S2). We demonstrate significant downregulation of BRD4 mRNA and protein levels (Supplemental Fig. S2*A–C*), albeit these changes are not as robust as observed in MC3T3 cells. However, as indicated by cell counting, most AMSCs undergo successful transfection as indicated by siGLO green transfection indicator (Supplemental Fig. S2*D, E*). Similar to MC3T3 cells (Fig. 6), when compared to Ad‐BMP2 transduced and siRNA control transfected cohorts, AMSCs lacking BRD4 expression that have been transduced with Ad‐BMP2 enhance alkaline phosphatase activity of naïve MC3T3 cells undergoing osteogenic differentiation in transwell assay cultures (Fig. 7*B*) as well as conditioned medium cultures (Fig 7*C*). In support, Alizarin red staining, which detects hydroxyapatite deposition and is an established osteogenic assay, is significantly enhanced in naïve MC3T3 cell cultures grown in conditioned medium from AMSCs transduced with Ad‐BMP2 with suppressed BRD4 expression (Fig. 7*D*). It is important to point out that naïve MC3T3 were exposed to the conditioned medium for the first 9 days (days 0 to 9, three media changes), while Alizarin red staining occurred on day 27 of osteogenic differentiation, suggesting that a relative short exposure to enhanced BMP2 levels can have a dramatic impact on osteogenic differentiation within the local environment. Thus, similar to MC3T3 cells, depletion of BRD4 can enhance production of adenoviral BMP2 in AMSCs to stimulate osteogenic differentiation within the local environment.

## Discussion

Our present studies demonstrate that Brd4 depletion suppresses osteogenic differentiation of primary BMSCs. Our findings are supported by previous studies which demonstrated that BET protein inhibition (ie, +JQ1) and Brd4 depletion inhibit osteogenic differentiation of established immortalized cell systems, including MC3T3‐E1 pre‐osteoblasts, IDG‐SW3 osteocytes, as well as human fetal osteoblasts.^(^ ^30^, ^37^ ^)^ Mechanistically, it is predicted that Brd4 is recruited to regulatory regions within the genome to promote lineage‐specific gene expression and direct differentiation of mesenchymal progenitor cells to a committed and mature state that supports tissue homeostasis.^(^ ^30^, ^37^, ^38^ ^)^ Permanent Brd4 depletion or long‐term BET protein inhibition halts osteogenesis, but osteogenic lineage commitment can be restored after short‐term blockade of BET proteins. On the other hand, early and short‐term inhibition of histone code writers (eg, Ezh2) and erasers (histone deacetylases) generates a lasting epigenetic effect (alteration of epigenetic memory or epigenetic priming) that alters phenotype of differentiating progenitor cells.^(^ ^21^, ^26^, ^42^, ^50^, ^51^, ^52^ ^)^ These findings are in line with the idea that inhibiting the activities of histone code writers and erasers changes the epigenetic landscape, while the targeting of BET proteins prevents their binding to preexisting chromatin states to support the transcriptional machinery.^(^ ^26^, ^37^, ^51^, ^53^ ^)^

In addition to altering the osteogenic phenotype of BMSCs, our studies also reveal that depletion of Brd4 enhances expression of adenoviral transgenes in several cell models including mouse BMSCs, mouse MC3T3‐E1 cells, and human AMSCs. We also demonstrate that BET protein inhibition elevates adenoviral transgene expression. While our studies were under investigation, Lv and colleagues demonstrated that BET inhibition also enhances adenoviral transgene expression in mammalian cells.^(^ ^54^ ^)^ Their studies suggest that enhanced effects require the presence of Brd4 protein, while our present studies demonstrate that Brd4 protein reduction stimulates expression of adenoviral transgenes. We acknowledge that only modest depletion of Brd4/BRD4 is observed in our in vitro models, which includes depletion of this epigenetic regulator via Ad‐Cre transduction in Brd4^fl/fl^ BMSCs and siRNAs‐mediated depletion in MC3T3 and AMSCs. Further investigation into the mechanisms underlying this effect are needed and may provide additional insight into this divergence in findings. Nonetheless, our results clearly show in three separate cell culture models and different experimental modulations that BET inhibition and Brd4 protein depletion stimulates adenoviral gene expression.

Because our studies utilized adenoviruses that are unable to integrate into the host genome and are replication deficient, the enhanced adenoviral transgene expression could be attributed to enhanced viral entry, transcription, or translation. The most direct interpretation is that enhanced mRNA expression of three different types of viral transgenes (ie, GFP, Cre, and BMP2) observed in our studies is due to increased viral vector‐based transcription. Previous studies suggest that Brd4 may directly inhibit lentiviral transcription based on interactions with viral specific components.^(^ ^55^, ^56^ ^)^ Links between acetylation and viral transcription are evident from studies showing that inhibition of histone deacetylases (which increases genomic chromatin acetylation) enhances the oncolytic effects of adenovirus infection.^(^ ^57^, ^58^, ^59^ ^)^ While the latter appears to occur through transcription and not transduction‐based mechanisms,^(^ ^60^ ^)^ the complexity of adenoviral oncolysis does not permit a straightforward mechanistic comparison with our current work. Alternatively, viral transcription may very well also be favored by Brd4 depletion or inhibition because the transcriptional machinery may no longer be stabilized by Brd4 on the host genome and therefore could be diverted to the viral genome. In this model, eliminating Brd4 protein or inhibiting its activity with BET inhibitors would decrease Brd4 occupancy of the host genome and allow loading of non‐integrated viral DNA with the transcriptional machinery that is released from the host genome. The translational relevance of reducing viral vector loads while maintaining high transgene expression warrants future mechanistic studies to validate this putative mechanisms by which BET‐related proteins and histone acetylation may influence viral vector expression in mammalian cells.

Viral vector gene therapy is a growing area of research and is gaining popularity as an alternative therapeutic strategy. As such, a wide array of viruses and viral vectors have been engineered and tested in clinical trials.^(^ ^61^, ^62^ ^)^ Viral‐mediated delivery of BMP2 shows promise as a therapeutic alternative to local delivery of high BMP2 concentrations.^(^ ^61^ ^)^ Translational studies have demonstrated that MSCs transduced with Ad‐BMP2 restore the bone healing process in critical‐sized defect animal models.^(^ ^63^, ^64^ ^)^ Mechanistically, studies suggest that the transduced and implanted MSCs serve as the BMP2 delivery vehicle and may not directly contribute to the healing process (ie, do not become the bone‐building osteoblasts).^(^ ^65^ ^)^ Although promising, adenoviral delivery of BMP2 is partially limited by the immune response to high viral loads and concern regarding the persistence of BMP2 expression within the defect.^(^ ^61^ ^)^ Thus, strategies that can reduce the viral load and deliver adequate amounts of BMP2 may provide clinical usefulness.

Our present study provides proof‐of‐concept that adenoviral transgene expression can be significantly enhanced by targeting of Brd4 in multiple cell models without increasing the viral load. Our studies with adipose tissue–derived MSCs are especially encouraging as cell therapy has gained traction in the treatment of disease.^(^ ^45^, ^66^, ^67^, ^68^, ^69^ ^)^ Although initial studies utilizing MSCs were based on the idea that these cells may become a part of the healing tissue, recent evidence suggests the therapeutic effect of these cells may stem from their trophic functions within the local environment.^(^ ^70^, ^71^, ^72^, ^73^ ^)^ While other cell‐based therapies (eg, induced pluripotent and bone‐marrow MSCs) could be considered, the use of adipose tissue–derived MSCs to deliver adenoviral cytokines, including BMP2, is practical because cells can be obtained in large quantities and in a relatively painless and non‐invasive procedure.^(^ ^74^ ^)^

In summary, we establish that Brd4 is required for osteogenic commitment of BMSCs, but reduction or inhiition of Brd4 /BRD4 enhances the expression of exogenous adenoviral transgenes. Adenoviral BMP2 expression can be enhanced in transduced mesenchymal cells by Brd4 depletion. Importantly, the enhanced production of BMP2 by Brd4‐depleted cells supports osteogenic differentiation within the local environment by paracrine mechanisms. We envision scenarios in which biologics (eg, BMP2) are delivered in adipose tissue–derived MSCs that lack Brd4 expression to improve clinical outcomes that may be currently hindered by bioavailability of these biologics and toxicities that are associated with high viral titers.

## Disclosures

All authors state that they have no conflicts of interest.

## Supporting information


**Appendix S1.** Supplementary Information
