# Supplementary material for: Brd4 Inactivation Increases Adenoviral Delivery of BMP2 for Paracrine Stimulation of Osteogenic Differentiation as a Gene Therapeutic Concept to Enhance Bone Healing
Source: JBMR Plus. 2021 Jun 23;5(10):e10520. doi: 10.1002/jbm4.10520 (PMC8520065; doi:10.1002/jbm4.10520)
Supplement: Supplementary file 1 — Appendix S1. Supplementary Information [file JBM4-5-e10520-s001.docx]

**SUPPLEMENTAL MATERIAL**

**Brd4 inactivation increases adenoviral delivery of BMP2 for paracrine stimulation of osteogenic differentiation as a gene therapeutic concept to enhance bone healing**

Christopher R. Paradise, Ph.D.^1,2^, Rodolfo E. De La Vega^3,4,5^, M.D., M. Lizeth Galvan, D.D.S.^1^, Margarita E. Carrasco, Ph.D.^1^, Roman Thaler, Ph.D.^1^, Andre J. van Wijnen, Ph.D.^1,2,6*^, Amel Dudakovic, Ph.D.^1,6*^

^1^Department of Orthopedic Surgery, Mayo Clinic, Rochester, MN, USA

^2^Center for Regenerative Medicine, Mayo Clinic, Rochester, MN, USA

^3^Musculosketal Gene Therapy Research Laboratory, Rehabilitation Medicine Research Center, Mayo Clinic, Rochester, MN, USA

^5^Department cBITE, MERLN Institute for Technology-Inspired Regenerative Medicine, Maastricht University, Maastricht, the Netherlands

^5^Department IBE, MERLN Institute for Technology-Inspired Regenerative Medicine, Maastricht University, Maastricht, the Netherlands

^6^Department of Biochemistry and Molecular Biology, Mayo Clinic, Rochester, MN, USA

*Corresponding Authors:

Amel Dudakovic, Ph.D. ([Dudakovic.Amel@mayo.edu](mailto:Dudakovic.Amel@mayo.edu), 507-293-0105)

Andre J. van Wijnen, Ph.D. ([vanWijnen.Andre@mayo.edu](mailto:vanWijnen.Andre@mayo.edu), 507-293-2105)

Running Title: Brd4 and adenoviral gene expression

**Supplemental Table 1: RT-qPCR primers used in this study.**

| **GENE ID** | **FORWARD PRIMER** | **REVERSE PRIMER** |
| --- | --- | --- |
| **Gapdh** | CATCACTGCCACCCAGAAGACTG | ATGCCAGTGAGCTTCCCGTTCAG |
| **Sp7** | GGCTTTTCTGCGGCAAGAGGTT | CGCTGATGTTTGCTCAAGTGGTC |
| **Bglap** | GCAATAAGGTAGTGAACAGACTCC | CCATAGATGCGTTTGTAGGCGG |
| **Ibsp** | GAATGGCCTGTGCTTTCTCG | CCGGTACTTAAAGACCCCGTT |
| **Alpl** | CCAGAAAGACACCTTGACTGTGG | TCTTGTCCGTGTCGCTCACCAT |
| **GFP** | CACATGAAGCAGCACGACT | GGTCTTGTAGTTGCCGTCGT |
| **Cre** | ACCAGCCAGCTATCAACTCG | TTACATTGGTCCAGCCACC |
| **Brd4** | GGAGGAAAGAAACAGGGGCA | GAGTCTGAAGTGGCTGAGGG |
| **Phospho1** | ATGAGCGGGTGTTTTCCAG | TGCCGTCCCTAGATAGGCATC |
| **BMP2** | GCTGTCTTCTAGCGTTGCTG | CTGTTTCAGGCCGAACATGC |

**Supplemental Figure 1. Transfection with Brd4 siRNAs effectively reduces Brd4 protein levels in MC3T3-E1 cells.** MC3T3-E1 cells were transfected with control or Brd4 siRNA. Brd4 protein levels were assessed by Western blotting (n = 3, mean ± standard deviation) (**A&B**) and Brd4 mRNA expression was evaluated by RT-qPCR analysis (n = 3, mean ± standard deviation) (**C**) 48 hour after transfection. MC3T3 cells were transfected with siGLO (green) and stained with Hoechst (blue) 24 hours later to assess transfection efficiency (**D**). Subsequent ImageJ analysis of overlap of siGLO and Hoechst staining provides assessment of transfection efficiency (n = 3, mean ± standard deviation) (**E**). Points on the graph represent biological replicates. Statistical significance is indicated in the figure panels.


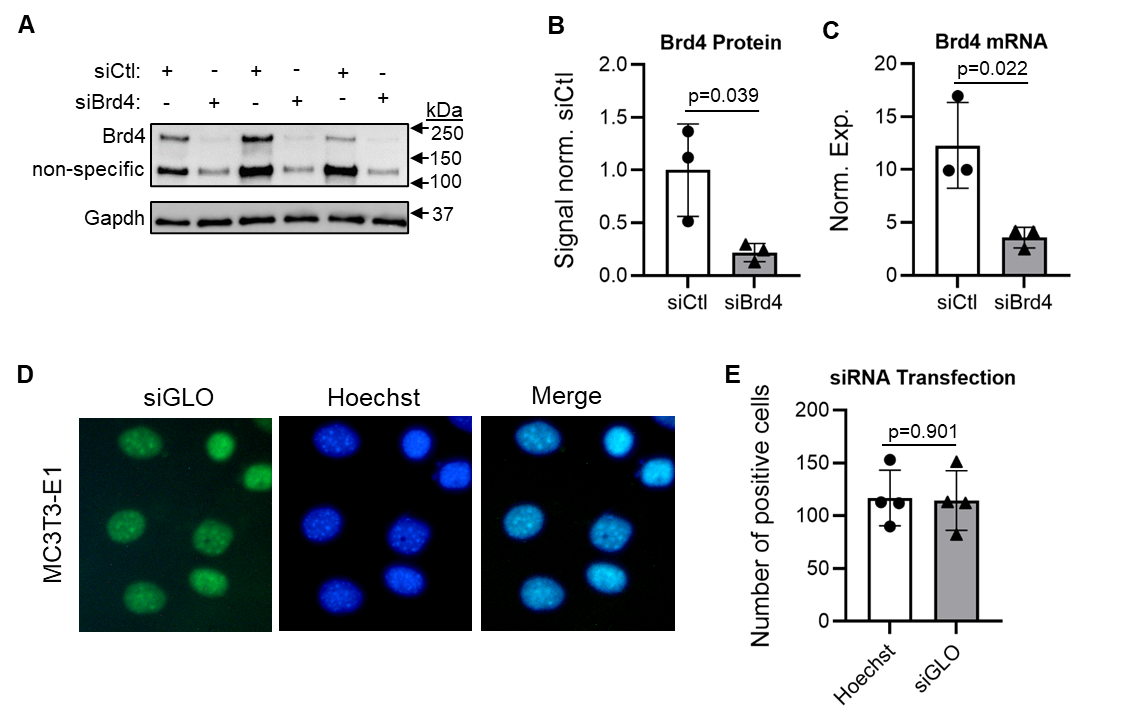


**Supplemental Figure 2. Transfection with BRD4 siRNAs effectively reduces BRD4 protein levels in AMSCs.** AMSCs were transfected with control or BRD4 siRNA. BRD4 protein levels were assessed by Western blotting (n = 3, mean ± standard deviation) (**A&B**) and BRD4 mRNA was evaluated by RT-qPCR analysis (n = 3, mean ± standard deviation) (**C**) 48 hour after transfection. AMSCs were transfected with siGLO (green) and stained with Hoechst (blue) 24 hours later to assess transfection efficiency (**D**). Subsequent ImageJ analysis of overlap of siGLO and Hoechst staining provides assessment of transfection efficiency (n = 3, mean ± standard deviation) (**E**). Points on the graph represent biological replicates. Statistical significance is indicated in the figure panels.


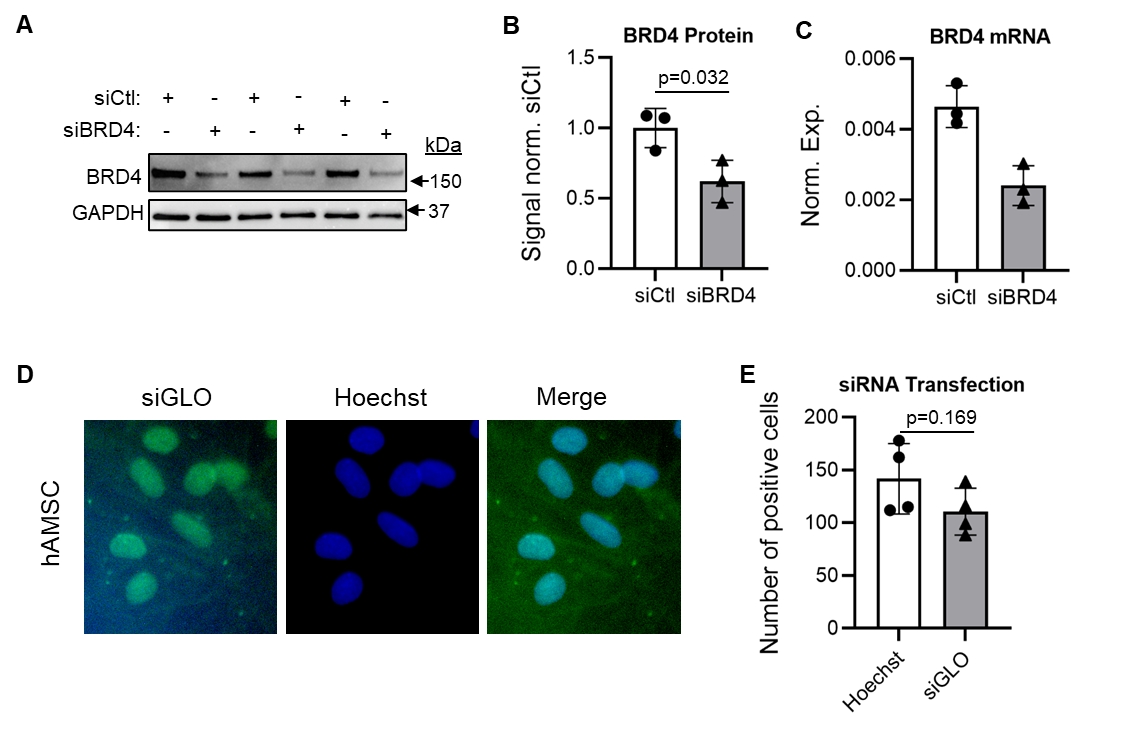


**Original Western Blot Images:**

**Figure 2B**

**
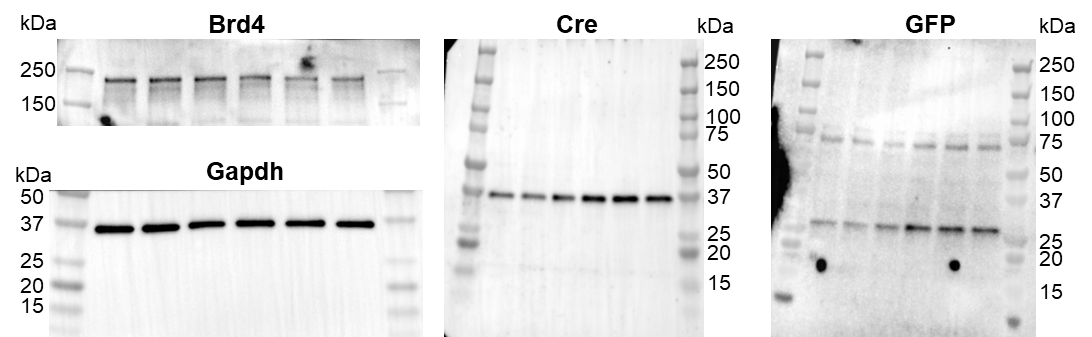
**

**Figure 2C**

**
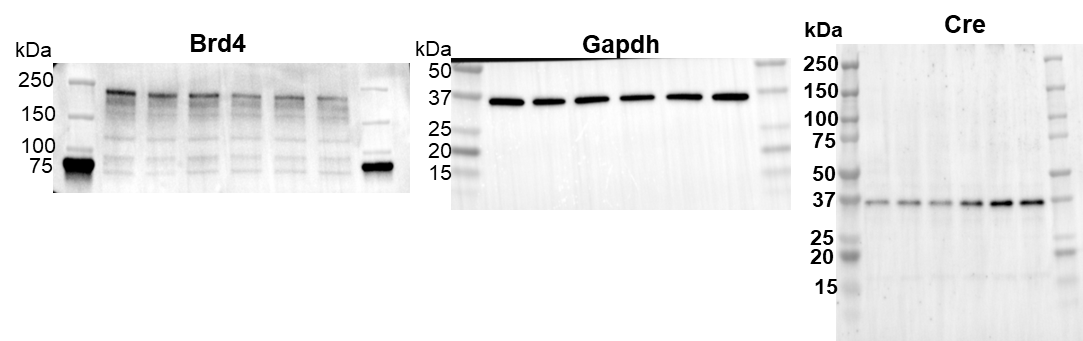
**

**Figure 3B** (Blots for Brd4, GFP, and Cre are outlined in dotted red box).

**
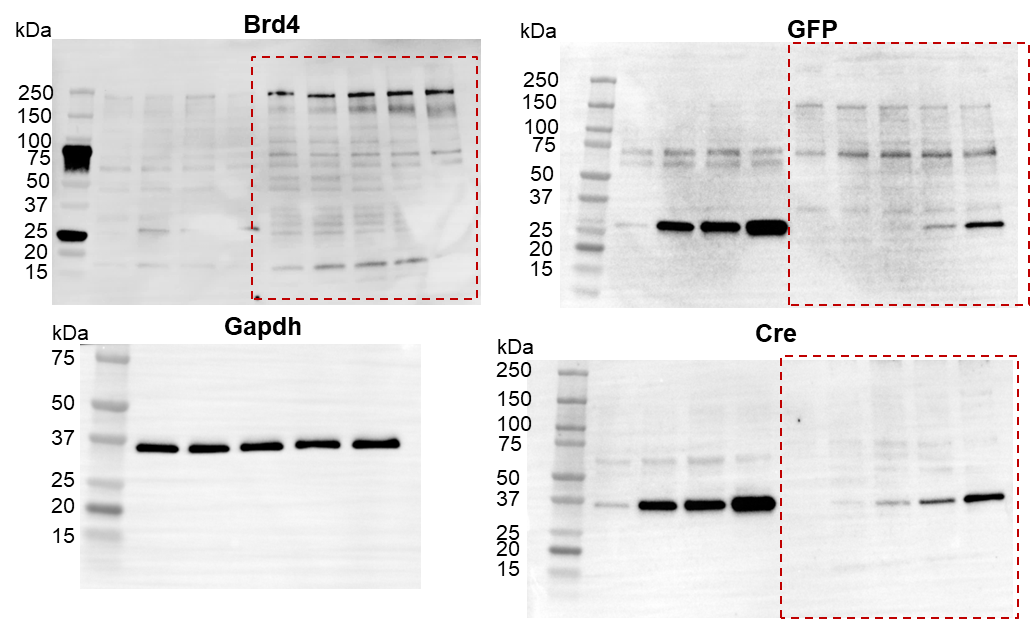
**

**Suppl Fig 1A**

**
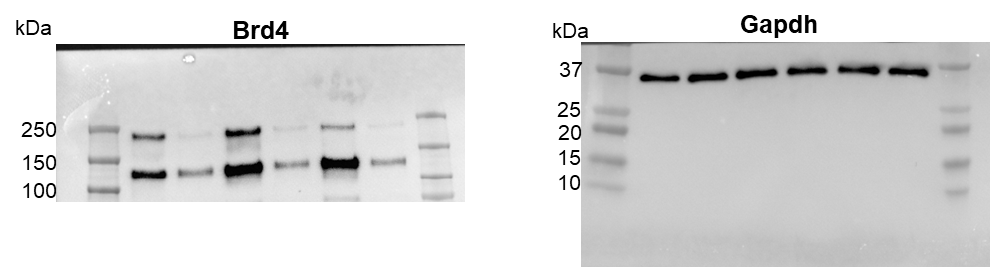
**

**Supp Fig 2A**

**
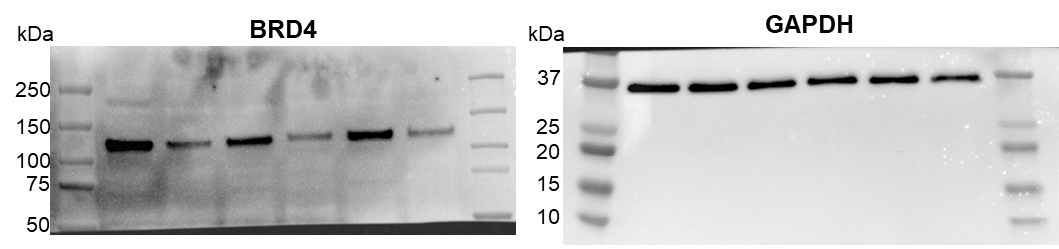
**
